# New Approaches in Hypertension Management: a Review of Current and Developing Technologies and Their Potential Impact on Hypertension Care

**DOI:** 10.1007/s11906-019-0949-4

**Published:** 2019-04-25

**Authors:** Jamie Kitt, Rachael Fox, Katherine L. Tucker, Richard J. McManus

**Affiliations:** 1 Radcliffe Department of Medicine (Cardiovascular Division), University of Oxford, Oxford, UK; 2 Nuffield Department of Primary Care Health Sciences, University of Oxford, Radcliffe Primary Care, Radcliffe Observatory Quarter, Woodstock Road, Oxford, OX2 6GG UK

**Keywords:** Self-monitoring, Hypertension, Smartphones, Apps, Tele-monitoring, Self-management

## Abstract

**Abstract:**

Hypertension is a key risk factor for cardiovascular disease. Currently, around a third of people with hypertension are undiagnosed, and of those diagnosed, around half are not taking antihypertensive medications. The World Health Organisation (WHO) estimates that high blood pressure directly or indirectly causes deaths of at least nine million people globally every year.

**Purpose of Review:**

In this review, we examine how emerging technologies might support improved detection and management of hypertension not only in the wider population but also within special population groups such as the elderly, pregnant women, and those with atrial fibrillation.

**Recent Findings:**

There is an emerging trend to empower patients to support hypertension screening and diagnosis, and several studies have shown the benefit of tele-monitoring, particularly when coupled with co-intervention, in improving the management of hypertension.

**Summary:**

Novel technology including smartphones and Bluetooth®-enabled tele-monitoring are evolving as key players in hypertension management and offer particular promise within pregnancy and developing countries. The most pressing need is for these new technologies to be properly assessed and clinically validated prior to widespread implementation in the general population.

## Introduction

Hypertension has been identified by WHO [1] as one of the most significant risk factors for morbidity and mortality worldwide and is responsible for the deaths of approximately nine million people annually [1]. In the UK, the National Institute for Health and Care Excellence (NICE) [2] defines high blood pressure (BP), also known as hypertension, as a clinic blood pressure of 140/90 mmHg or higher confirmed by a subsequent ambulatory blood pressure monitoring daytime average (or home blood pressure monitoring average) of 135/85 mmHg or higher.

High blood pressure does not just develop in older adults. Over 2.1 million people under 45 years old had high blood pressure in England in 2015 [3]. This is important because treating hypertension results in significant reductions in risk of subsequent cardiovascular disease [4, 5]. Despite strong evidence for such treatment, studies suggest that many people remain sub-optimally controlled [6]. New approaches, including new technologies, are therefore needed to improve screening, detection and control of raised blood pressure in the community.

## Screening

High blood pressure is largely asymptomatic, especially in the early stages, leading to its description as a ‘silent killer’ [1]. The asymptomatic nature of hypertension in conjunction with its disease burden necessitates routine blood pressure screening. In the UK, NICE guidelines recommend blood pressure measurement at least yearly among normotensive adults [3] and currently hypertension is largely identified in this way by physicians routinely or opportunistically assessing blood pressure in a primary care clinic setting [7]. However, it has been estimated that between a third and a half of hypertensive patients remain undiagnosed, indicating the need for better screening [8]. Developments in non-physician-based blood pressure measurements utilising new technologies may provide an opportunity for increased detection of hypertension.

Self-screening allows patients to measure their own blood pressure outside of physician consultations, either in their own home or with public validated solid cuff automatic sphygmomanometers that require no training, just simple instructions for use [7]. In Japan, the market penetration of home blood pressure monitoring is such that it is estimated that more than enough monitors have been sold for one per household. In the UK, at least 1:10 normotensive adults have measured their own blood pressure at some time in the past [9]. A recent systematic review [7] identified three studies of self-screening, which utilised public blood pressure cuffs in a variety of settings including pharmacies and grocery stores (Hamilton 2003 [10], Houle 2013 [11], Nykamp 2016 [12]). The majority of these were conducted in North America, where out-of-office blood pressure self-screening stations in pharmacies and work places are estimated to be used more than one million times a day [13]. Providing additional blood pressure self-monitoring equipment in physician waiting rooms has been proposed in the UK to increase blood pressure screening [14], and such monitors are available in around a third of practice in the UK [15]. Whilst several studies to date show promising results for feasibility, patient autonomy, convenience, and increased detection of hypertension (Hamilton 2003 [10], Houle 2013 [11] and Tompson 2017 [14]), a number of barriers are yet to be overcome before widespread community self-screening can be recommended. These include limited privacy, poor awareness of the availability of the facilities, and a lack of education regarding the asymptomatic nature of hypertension and the benefits of screening [14].

Breaking away from traditional cuff-based measurement of blood pressure, the widespread accessibility of smartphones and mobile health applications also offer new potential for the ubiquitous monitoring of parameters such as blood pressure. Recently, for example, the Cardiogram® application on the Apple® watch has been evaluated for its utility at using deep learning algorithms to predict hypertension from inputs of heart rate and step count. Data were collected from 6115 app users for an average of 9 weeks and predicted hypertension moderately well [16]. This particular ‘app’ can now utilise multiple other wearable devices such as Fitbit®, Garmin® and Android devices; however, further research into its diagnostic utility is required. Furthermore, in the UK at least, current market penetration of smartphones into elderly populations is not sufficient for these techniques to be widely available in this key age group, but they have definite potential to aid detection of hypertension in younger adults [Ofcom communications market 2018]. In addition, cognitive deficits and visual or hearing impairments, which are more prominent in the older population, can decrease the accessibility of smartphone applications. It seems likely that further advances in technology will increase the spread of such techniques, but the need for long-term treatment of hypertension means that a formal diagnosis of hypertension is likely to remain paramount.

## Hypertension Diagnosis

Once a person has been screened and found to have high blood pressure, ambulatory blood pressure monitoring (ABPM) is regarded as the most accurate way to diagnose hypertension and is recommended by guidelines to routinely to confirm elevated blood pressure readings [2, 17, 18]. Ambulatory monitors typically involve portable, automated cuffs worn continuously that measure blood pressure every 15–30 min during the day and 15–60 min overnight [19]. Despite their utility in diagnosis, ambulatory monitors may not be available to many clinicians and patients due to cost and time limitations [19] and can be uncomfortable and disruptive to daily life and sleep [9, 20]. Advances in technology have allowed for the development of new ‘cuff-less’ BP monitoring devices however, which continuously monitor BP without disruption to daily activities. Cuff-less BP monitoring devices utilise smartphone or wearable sensor technologies that can estimate BP from ECG signals, photoplethysmogram (PPG) signals (using infrared light on the finger to estimation of skin blood flow), or a combination of both [21]. For example, one system developed consists of a wearable wrist band to collect PPG signals, a wearable heart rate belt to collect ECG signals, and a smartphone. The signals from the wearable device communicate via Bluetooth with the smartphone to synchronise their measurements and continuously stream the wearer’s blood pressure. Other devices that have been developed utilise sensors in T-shirts [22], placed behind the ear [23] and in a computer mouse [24] to calculate and record blood pressure measurements.

As with screening, the use of ‘smartphone apps’ is increasingly popular to aid in diagnosis. One US survey of ‘app users’ showed that 31% of mobile phone owners used their phone to look for health information, with the largest proportion (52%) among smartphone users [25]. Although this is a hugely expanding field, with > 180 apps now existing to measure blood pressure, in only 3.8% (7/184) of the blood pressure apps was any involvement of medical experts mentioned in its development and very few apps have been robustly evaluated [25]. Moreover, at present, no mobile apps have formally obtained approval for use as measuring/diagnostic devices by the US Food and Drug Administration or European Commission. The American Heart Association (AHA) has stated that there are too many errors with smartphone blood pressure apps [26] with mobile app–based blood pressure measurements being inaccurate four out of five times when one popular mobile application was tested [25, 26].

A vital issue with both the apps and novel non-invasive devices is the lack of a universally agreed standard for the validation of this technology, and current protocols simply do not include them. There are plans to rectify this [27•] with some apps exploring clinical validation [28, 29] so the future does look brighter. At present, however, there is limited incorporation of this technology into widespread clinical practice as a result of this key issue [26].

## Hypertension Management

Around 14% of the adult population in England and Wales currently appear on primary care hypertension registers [8] which equates to over seven million people. This provides a significant market for technology to assist in control. Currently, 60% of those on hypertensive registers are controlled [30], and only 50% of those starting on a new antihypertensive remaining taking it after 6 months [3]. In this cohort of people, the technology to facilitate management has been available for some years but has only recently acquired a solid evidence base. Options considered in this section range from self-monitoring and tele-monitoring to virtual clinics and artificial intelligence (AI)–assisted management.

1. Self-monitoring of blood pressure can improve blood pressure control and is an increasingly common part of hypertension management. It is well tolerated by patients and has been shown to be a better predictor of end organ damage than clinic measurement [2, 20, 32, 33]. Trials of self-monitoring show improved blood pressure control, mainly in the context of additional co-interventions such as pharmacist intervention or nurse-led education [34]. A caveat to self-monitoring is that it relies on good communication between patients and physicians, and perhaps 50% of patients do not tell clinicians they are self-monitoring or share the readings with their physician, in a meaningful manner [35]. A solution to this may be the remote monitoring of blood pressure readings measured at a patient’s home, i.e. tele-monitoring, something explored more below.

Another option to enhance ongoing self-monitoring compliance could be BP monitoring apps. These can communicate between smartphone and BP monitor allowing the patient to control (e.g. start/stop/configure) the BP measurement procedure from the app and to download automatically the current or previous BP readings. BP estimation is computed in the device microchip using the oscillometric signal, which is sampled and filtered from device pressure sensors, during the cuff inflation or deflation. Examples of BP self-monitoring analytics subsequently available include tracking the average BP over time, alerting on concerning BP trends, e.g. high/low readings, or normal/abnormal circadian BP patterns (dipper/non-dipper trend). When an app is used to communicate with a clinician, this becomes a type of tele-monitoring (see below).

Self-monitoring can also be combined with self-titration of medication, a process known as self-management. Trials undertaken before the current generation of mobile devices have shown that self-management can lead to improved blood pressure control through medication optimisation in both hypertensive and higher risk populations [36•, 37•].

2. Tele-monitoring is a particular application of telemedicine—the transfer of data remotely—which in this case consists of automatic data transmission of BP readings. It can also be combined with the transfer of other parameters such as heart rate, oxygen saturations, and pacemaker/defibrillator data from the patient’s home or workplace to a professional healthcare environment such as a primary care clinic/surgery or the hospital [38]. Several tele-monitoring systems are available which differ in their modality of data collection, transmission, and reporting and by the presence/absence of additional features such as reminders for BP measurement to be performed or medication reminders. Randomised controlled trials [39•] performed in recent decades have tested the effectiveness of home blood pressure tele-monitoring for the improvement of hypertension control and associated healthcare outcomes. In a large meta-analysis [39•], all studies included demonstrated a high degree of acceptance of the technologies by doctors and patients, good adherence to tele-monitoring programs and confirmed that the technology has the potential to enhance hypertension management, improve patient outcomes, and reduce healthcare costs, particularly when considering long-term follow-up.

Another meta-analysis demonstrated that BP tele-monitoring in conjunction with co-intervention, such as medication titration by a case manager or education/lifestyle counselling, led to significantly larger and persistent (up to 12 months) BP reductions when compared with self-BP monitoring alone without transmission of BP data and counselling [34].

Until recently, the key evidence missing from trials of self-monitoring and tele-monitoring was whether the use of such data by clinicians actually led to lower blood pressure. In 2018, the TASMINH4 trial [40•] showed that GPs using self-monitored blood pressure to titrate antihypertensives, with or without tele-monitoring, achieved better blood pressure control for their patients than those using clinic readings. As with previous trials, the mechanism of action appeared to be medication optimisation. The tele-monitoring group achieved lower blood pressure quicker than the self-monitoring group, but readings were not significantly different at the primary end point of 1 year. Forthcoming work shows that patient and clinician experience was largely positive from tele-monitoring with some important caveats in particular patients. Cost-effectiveness analysis suggests that self-monitoring in this context is cost-effective by NICE criteria, i.e. costing well under £20,000 per QALY [Grant S et al. BJGP 2019, *In Press; and* Monaghan M et al. Hypertension 2019, *In Press*].

Interactive digital interventions now offer the ability to provide users with additional support over and above simple tele-monitoring which can also result in lower blood pressure than usual care [41]. This can include, for example, multi-media demonstrations of lifestyle advice utilising video and web links. The ‘Home BP’ trial will report later in 2019 on the effectiveness of a web-based digital intervention with a lifestyle module testing the efficacy over and above usual care [42]. Where a digital intervention utilises mobile phone technology to underpin tele-monitoring, this is increasingly termed ‘M-health’.

3. ‘Virtual clinics/visits’ provide a system-level option for the use of such technology and comprise structured asynchronous online interactions between a patient and a clinician to extend medical care beyond the initial office visit. A study by Levine et al. in 2018 showed that for primary care patients managed for hypertension with a virtual visit vs. a real-life in-person visit, there was no significant adjusted difference in systolic blood pressure control, number of specialist visits, emergency department presentations, or inpatient admissions [43].
4. Other novel advances in hypertension management

Artificial intelligence underpins interfaces such as Alexa® and Siri® which can wirelessly update medication lists and set reminders (e.g. alarm reminders to take medications to improve adherence to treatment), and although there is a current dearth of evidence of the efficacy of these, it seems likely that their use will increase over time. Incorporation of tele-monitored data on blood pressure into digital healthcare programmes can now also allow combination with other physiological variables including blood glucose, heart rate and exercise allowing adaptation of management recommendations based on pre-determined variables including user demographics, indicated morbidities and comorbidities, self-identified barriers and actions recorded over the course of a programme or set by a physician. Examples of this include the ‘WellDoc Hypertension and diabetes management platform’ and ‘Omada Health’s digital program’.

## Implementation of Technology in Special Groups

Hypertension is an ideal area for the use of new technology but does require consideration of a number of special groups, the most important of which are discussed below:

### Atrial Fibrillation

Hypertension is a risk factor for atrial fibrillation (AF), and half of those with AF have hypertension [44], making blood pressure measurement an important aspect of care in these patients. However, the accuracy of current methods of blood pressure monitoring is limited in those with AF as demonstrated in a recent meta-analysis [45]. This is particularly an issue in the elderly where AF can affect over 10% of the population. Validation studies of automated blood pressure devices typically exclude those with AF, resulting in a lack of evidence regarding the accuracy of these devices to measure BP when AF is present, which is turn makes reliable out-of-office BP measurement, including home and ambulatory BP monitoring more difficult in this population. As a result, NICE [2] and European guidelines [17] currently both recommend manual measurement of blood pressure when AF is present, making self-monitoring very difficult [46]. A more recent systematic review analysed studies containing 14 different automated BP devices to determine if their accuracy in the presence of AF has improved as technology and detection algorithms have advanced [45]. In this study, of the devices compared, four were newer automated BP devices that incorporated the latest algorithms to detect AF, but the marketing for these devices appeared misleading as despite claiming ‘AF detection’ and ‘BP measurement’ within the same device, there was no evidence to suggest that they were more accurate at measuring BP in the presence of any atrial arrhythmia. This particular review [45] concluded that BP devices known to be accurate for patients in sinus rhythm cannot be assumed to maintain accuracy when used to measure BP in those with AF. Consequently, measurement, and thus management of BP, in patients with AF remains an area in which further development of new technology is required to enable more precise monitoring and management.

### Pregnancy

Hypertension in pregnancy results in substantial maternal morbidity and mortality worldwide [47, 48]. Furthermore, hypertension during pregnancy has been linked to the development of chronic hypertension and an increase in lifetime cardiovascular risk of at least double [49]. Self-monitoring of BP in pregnancy has been shown to be feasible and to have the potential to detect hypertensive disorders sooner than standard care [50]. Two large trials are currently recruiting (BUMP1 and BUMP2, https://clinicaltrials.gov/ NCT03334149) and aim to assess whether self-monitoring improves the detection and/or control of hypertension in pregnancy. Moreover, a recent feasibility trial of self-management of BP following hypertensive pregnancy [35] demonstrated that self-management using a purpose-designed app offers great promise in optimising post-partum BP management. This app allowed women to record self-monitored BP, to receive reminders to monitor their BP, and provided real-time automated medication titration feedback based on NICE guidance at that time [49] regarding self-titration and safety. Feasibility testing suggested that this technique was acceptable, as women self-monitored daily with 85% adherence and a median accuracy of 94% and there was a significant improvement in blood pressure control. This was most marked at 6 weeks, and interestingly, the difference in diastolic readings persisted to 6 months despite all but one woman finishing therapy [35]. These findings have prompted further follow-up of the women originally in this study and a larger, pilot study on self-management in the post-partum hypertensive cohort, both commencing later in 2019.

### Children

The first report on paediatric hypertension by the National Heart, Lung, and Blood Institute (NHLBI), published in 1977 [51] declared that “Detection and management of hypertension in children and the precursors of hypertension in adults are the next major frontier”. The report also recommended annual BP measurement in all children ≥ 3 years. Unfortunately, nearly 40 years later, the diagnosis of hypertension is missed in the majority of cases, and familiarity with paediatric hypertension among clinicians is extremely poor. This is therefore an area where the technology described above could make a real difference. However, the issues of validation of the technology are even more acute in the paediatric population because children’s vasculature and arm size are not the same as those of adults. The new universal standard provides recommendations aiming to improve this [27•].

### Developing Countries

New technology offers huge promise in low- and middle-income countries and is being embraced by projects such as CRADLE. This team have developed and validated several devices [52, 53] which were developed specifically to meet the World Health Organisation criteria for use in a low-resource setting. The newest device is low cost at approximately $20 per device, has low-power requirements, and can be charged using a standard mobile phone charger [54]. It is also robust and capable of accurately detecting abnormalities in vital signs, including during pregnancy [55]. Severe bleeding, severe infection, and blood pressure disorders [55] are the most common cause of deaths in pregnancy, and such devices have the potential to be life-saving. Resources are the biggest issue in the developing world however where many hospitals do not currently have appropriate monitoring equipment, let alone the newest technology.

## Future Research Needed

Whilst much has been achieved in terms of research to date, several areas are clearly lacking in the kind of evidence needed in primary and secondary care alike. The most pressing need is perhaps for new technologies to be assessed and clinically validated [27•] prior to widespread implementation in the general population.

As healthcare is moving towards greater patient involvement and responsibility, including self-monitoring and self-screening of hypertension, we need to understand how best clinicians and patients alike can integrate these advances into daily practice.

Much previous research around blood pressure monitoring and management has excluded those with additional or complex needs such as the very old, multi-morbid, or pregnant women. It is important to complete research in these populations, as there may be differences in accuracy in some groups [56, 57] and the implications of, for instance, white coat hypertension, may be very different in pregnancy compared with the general population.

## Conclusions

Hypertension has been identified by WHO as one of the most significant risk factors for morbidity and mortality worldwide [1], and despite strong evidence for treatment, studies suggest that many people remain sub-optimally controlled [6]. New approaches, including new technologies, are therefore needed to improve screening, detection and control of raised blood pressure in the community. Breaking away from traditional cuff-based measurement of blood pressure, the widespread accessibility of smartphones and mobile health applications offers new prospects for ubiquitous monitoring of parameters such as blood pressure, but evidence of both accuracy and efficacy is currently lacking.

Current market penetration of smartphones into the elderly is not sufficient for widespread implementation of technology such as smartphone apps in this age group, but M-health has definite potential to aid screening and diagnosis in younger adults, pregnant women, children and adolescents as well as older populations as the technology becomes more commonplace. A key issue with both apps and novel non-invasive devices are the lack of a universally agreed standard for the validation of this technology, and current protocols simply do not include them. There is thus limited incorporation of this technology into clinical practice at present [26], and this must be addressed as a matter of urgency by European, UK, and American regulators.

Until recently, the key evidence missing from trials of self-monitoring and tele-monitoring was whether the use of such data by clinicians actually led to lower blood pressure. Now trial data combined in meta-analyses provides strong evidence for BP tele-monitoring in conjunction with co-interventions, such as medication titration or education/lifestyle counselling. Further work is needed to ensure the most appropriate and beneficial aspects of technology are effectively utilised within the health system as this could improve care whilst reducing the need for face to face clinical appointments.
